# Supplementary material for: COZOID: contact zone identifier for visual analysis of protein-protein interactions
Source: BMC Bioinformatics. 2018 Apr 6;19:125. doi: 10.1186/s12859-018-2113-6 (PMC5889581; doi:10.1186/s12859-018-2113-6)
Supplement: Supplementary file 2 — Software build. Executable binary file of the software tool. (ZIP 118,784 kb) [file 12859_2018_2113_MOESM2_ESM.zip › cozoid/platform/docs/testng-6.5.1-javadoc.zip!/allclasses-frame.html]

All Classes (TestNG)


**All Classes**
  

AfterClass
  
AfterGroups
  
AfterMethod
  
AfterSuite
  
AfterTest
  
Assert
  
AssertJUnit
  
BeforeClass
  
BeforeGroups
  
BeforeMethod
  
BeforeSuite
  
BeforeTest
  
ClassMethodMap
  
CommandLineArgs
  
Configuration
  
ConversionUtils
  
Converter
  
DataProvider
  
DependencyMap
  
ExpectedExceptions
  
Factory
  
FileAssert
  
Guice
  
*IAnnotation*
  
*IAnnotationTransformer*
  
*IAnnotationTransformer2*
  
*IAttributes*
  
*IClass*
  
*IConfigurable*
  
*IConfigurationAnnotation*
  
*IConfigurationListener*
  
*IConfigurationListener2*
  
*IConfigureCallBack*
  
*IDataProviderAnnotation*
  
*IExecutionListener*
  
*IExpectedExceptionsAnnotation*
  
*IExtraOutput*
  
*IFactoryAnnotation*
  
*IHookable*
  
*IHookCallBack*
  
*IInstanceInfo*
  
*IInvokedMethod*
  
*IInvokedMethodListener*
  
*IInvokedMethodListener2*
  
*IMethodInstance*
  
*IMethodInterceptor*
  
*IMethodSelector*
  
*IMethodSelectorContext*
  
*IModuleFactory*
  
*IObjectFactory*
  
*IObjectFactory2*
  
*IObjectFactoryAnnotation*
  
*IParameterizable*
  
*IParametersAnnotation*
  
*IReporter*
  
*IResultMap*
  
*IRetryAnalyzer*
  
*ISuite*
  
*ISuiteListener*
  
*ISuiteResult*
  
*ITest*
  
*ITestAnnotation*
  
*ITestClass*
  
*ITestClassFinder*
  
*ITestContext*
  
*ITestListener*
  
*ITestMethodFinder*
  
*ITestNGListener*
  
*ITestNGListenerFactory*
  
*ITestNGMethod*
  
*ITestObjectFactory*
  
*ITestOrConfiguration*
  
*ITestResult*
  
*ITestRunnerFactory*
  
Listeners
  
NoInjection
  
ObjectFactory
  
Optional
  
Parameters
  
Reporter
  
ReporterConfig
  
ReporterConfig.Property
  
SkipException
  
SuiteRunner
  
SuiteRunnerWorker
  
SuiteRunState
  
Test
  
TestException
  
TestInstance
  
TestListenerAdapter
  
TestNG
  
TestNG.ExitCodeListener
  
TestNGAntTask
  
TestNGAntTask.Mode
  
TestNGAntTask.TestNGLogSH
  
TestNGException
  
TestNGUtils
  
TestRunner
  
TimeBombSkipException
  
XmlClass
  
XmlInclude
  
XmlMethodSelector
  
XmlPackage
  
XmlSuite
  
XmlTest
  
XmlUtils
